# Dynamic Relationships Between Perceived Housing and Life Transitions: A Better Understanding of Good Aging in Place

**DOI:** 10.1093/geroni/igab046.1715

**Published:** 2021-12-17

**Authors:** Frank Oswald, Steven Schmidt, Malcolm Cutchin

## Abstract

Housing has gained increased relevance as a central factor for health and well-being. Many countries have implemented ageing in place policies, which provide services focused on improving the physical environment. Housing needs change as people grow older and experience different transitions across their life courses. Studies have demonstrated relationships between housing and health and well-being in later life on the one hand and life transitions and health and well-being in later life on the other hand. However, research on life transitions in combination with perceived housing in relation to indicators of good ageing is virtually nonexistent. This symposiums aims to address the dynamic relationship between perceived housing and life transitions and how they impact health, well-being, functioning, and social/neighborhood participation as people age by data from a mixed-method approach in Sweden and Germany. The first contribution by Slaug and colleagues introduces changes in how older adults perceive their housing following the life transition of a fall at home. Second, Eriksson and colleagues present qualitative results on the experience of relationships between perceived housing, several life transitions and well-being among community-dwelling Swedish older adults. Third, Wanka and colleagues present partially different results from a comparable study in German on the same topic but emphasizing the experience of interrelationships between different life course transitions. Fourth, Granbom and colleagues explore how low-income older adults in Sweden reason about their current housing situation and a future life transition of relocation. Finally, Malcolm P. Cutchin will serve as the session’s discussant.

